# *SE*(3) group convolutional neural networks and a study on group convolutions and equivariance for DWI segmentation

**DOI:** 10.3389/frai.2025.1369717

**Published:** 2025-02-28

**Authors:** Renfei Liu, François Lauze, Erik J. Bekkers, Sune Darkner, Kenny Erleben

**Affiliations:** ^1^Department of Computer Science, University of Copenhagen, Copenhagen, Denmark; ^2^Department of Computer Science, University of Amsterdam, Amsterdam, Netherlands

**Keywords:** geometric deep learning, group action, homogeneous spaces GCNN, image segmentation, diffusion weighted imaging

## Abstract

We present an *SE*(3) Group Convolutional Neural Network along with a series of networks with different group actions for segmentation of Diffusion Weighted Imaging data. These networks gradually incorporate group actions that are natural for this type of data, in the form of convolutions that provide equivariant transformations of the data. This knowledge provides a potentially important inductive bias and may alleviate the need for data augmentation strategies. We study the effects of these actions on the performances of the networks by training and validating them using the diffusion data from the Human Connectome project. Unlike previous works that use Fourier-based convolutions, we implement direct convolutions, which are more lightweight. We show how incorporating more actions - using the *SE*(3) group actions - generally improves the performances of our segmentation while limiting the number of parameters that must be learned.

## 1 Introduction

In this work, we study the influence of group actions on data and how they may impact the architecture and performances of neural networks, especially convolutional neural networks (CNN). CNNs rely on assumed translational symmetries in data and have shown very robust performance in imaging tasks, especially medical imaging ones, and they are highly parameter-efficient due to their weight-sharing property. When data offer more structure than simply translation, this can be used to build generalized CNNs. This is especially the case for the task at hand—classification and segmentation of Diffusion Weighted Imaging (DWI) data. These Group and Geometric CNNs (GCNN) have been studied intensively and applied in many situations in the few past years (Masci et al., 2015; Cohen and Welling, 2016a; Boscaini et al., 2016; Bekkers et al., 2018; Cohen et al., 2020 to cite a few).

DWI is a non-invasive image modality that provides local information about water diffusion in tissues by means of measuring spin displacement (Tuchs, 2004). It provides three-dimensional diffusion information at each location *x* that can be encoded as a function *f*_*x*_ on the two-dimensional sphere *S*^2^. A field of these functions, on a given domain, can be represented as a function *f*:ℝ^3^×*S*^2^ → ℝ. If a sample is rotated and translated, the acquired signal should reflect, up to the limitations of acquisition protocol, this transformation. The group in question is the group of 3D rigid motions, *SE*(3), and the space ℝ^3^×*S*^2^ is a *homogeneous space* under the action of *SE*(3): A point in ℝ^3^×*S*^2^ can be transformed in any other point by a rigid transformation. This notion of homogeneous space is at the heart of the extension of CNNs to GCNNs (Cohen et al., 2020; Bekkers, 2019).

Our task at hand is the classification/segmentation of diffusion data. The inductive bias provided by the knowledge of these transformations may prove important for our task, especially when the amount of annotated data is limited. The problem boils down to how to incorporate this knowledge. The most classical approach is to use data augmentation, reflecting the expected symmetries in the data, in the hope that the network will be able to learn it during the training phase, learning symmetry-aware kernels.

Incorporating, on the other hand, some information about the symmetries of the data in the model has been shown to boost the performances of these networks (Bekkers et al., 2018). But how much of this information is needed for a given task? To provide an answer, for the DWI segmentation task, we propose several networks, which gradually incorporate these symmetries in their architecture and study their performances. In addition, instead of performing convolution on non-Euclidean data in a spectral fashion using Fourier-type transformations, we implement convolution in all our experiments in a direct way, as is usually done in the image analysis community. In other words, we use regular representations of groups to encode the group actions in the models, instead of irreducible representations. Our experiments, in some sense, perform a *group action ablation study*. We start with a “naive” CNN and then incorporate spherical symmetries, resulting in a *SO*(3)-GCNN, discarding the spatial aspect of the data. The spatial aspect is then added in the form of a standard CNN coupled with spherical symmetries, and then, we build a network where roto-translational transformations are used in almost all steps. This work demonstrates empirically the improvement in performance. The results are, however, not always clear-cut. Previous works associated with group convolutions have addressed the capabilities of their models in comparison with data augmentation but, to the best of our knowledge, have not touched the comparison between models tested under randomly transformed test sets. This is what our ablation study is providing. It not only shows the impact of embedding transformations in the model but also gives a systematic analysis on the comparison among different group actions and the corresponding elements of network architecture with respect to the interplay between rotations and translations - the physically justified roto-translation group and the simpler direct product of translations and rotations - imposed in the models, and their relation to data augmentation, both in the training and test set. In the study we provide, the GCNN built from 3D-translations on one hand and rotations, on the other hand, seems to perform better than a *SE*(3)-GCNN. However, the *SE*(3)-network generalizes better to unseen rotated data than the previous one. The reason may lie in the particular type of data used - our DWI scans come from the Human Connectome Project (HCP) (Van Essen et al., 2013) are highly preprocessed, including a form of alignment – and this may impact the results. Nevertheless, for every model we propose, we also experiment training them with data augmentation to compare with our equivariant networks. We show that the more equivariance we incorporate into the model, the better the model resists the inconsistency of distributions between training and testing data.

The contribution of this work is as follows.

- We extend the prior work (Liu et al., 2022) with a detailed theoretic formulation of the proposed method. We discretize *SO*(3) using the icosahedral rotation group and use rotation-translation separable filters in our model to make it very lightweight while achieving highly robust performance.
- We provide an ablation study of different group actions in different spaces and the combinations of these actions with additional experiments using data augmentation.
- We provide a comparison to Müller et al. (2021) in the experiments, which, to our knowledge, is the only other existing work that does tissue classification from DWI data using SE(3) group convolutions. In addition, we further provide experiments using the non-NN method of Schnell et al. (2009), which relies on rotationally invariant spherical harmonic (SH) features extracted from individual DWI voxels (squared-norms at given SH-orders), with classification performed by support vector machines (SVM). The spatial information is, however, discarded.

In the rest of this paper, we review related work, both around CNN and DWI classification problem. Then, we introduce the theoretical setup of GCNN and build several networks. Thereafter, we study and discuss their performances. Our implementation and experiments are publicly available at https://github.com/rliu-p/se3gcnn.

## 2 Related work

Deep Learning (DL) for non-flat data, or using more complex group actions than just translations, is currently getting more attention from the research field. When it comes to non-flat data, such as the point-wise spherical signals in DWI, particularly relevant related works are the following. A non-rotationally invariant modification was proposed by Boscaini et al. (2016). Schnell et al. (2009) developed an Support Vector Machine (SVM) using rotation-invariant features extracted from Spherical Harmonic decomposition of the HARDI signals, while Skibbe and Reisert (2017) introduced a toolkit for 3D image processing based on Spherical Tensor Algebra (STA), which is particularly well-suited for tasks requiring rotational invariance, such as image enhancement, reconstruction, and feature detection.

The above provide methods for DL-based processing of data on arbitrary manifolds. When the manifold, however, is a homogeneous space, i.e., there is a group action by which any two points on the manifolds can be reached, theory simplifies via a natural generalization of classical convolutions in group convolution neural networks (GCNNs), as was presented in Cohen et al. (2018); Bekkers et al. (2018); and Kondor and Trivedi (2018). GCNNs guarantee global equivariance. However, global equivariance can be complicated and elusive when the underlying geometry is non-trivial, which was discussed in Cohen et al. (2019). An elementary construction on a general manifold is proposed by Schonsheck et al. (2018) via a fixed choice of geodesic paths used to transport filters between points on the manifold, ignoring the effects of path dependency, i.e., holonomy when paths are geodesics. The removal of this path dependency can be obtained by summarizing local responses over local orientations, which is what was done by Masci et al. (2015). To explicitly deal with holonomy, Sommer and Bronstein (2020) proposed a theoretical breakthrough using convolution construction on manifolds based on stochastic processes via the frame bundle.

On the other hand, Cohen et al. (2018) lifted spherical functions to the 3D-rotation group *SO*(3) and used a generalization of Fourier transform on it to perform convolution. Elaldi et al. (2021) proposed an equivariant spherical deconvolution method to learn the orientation distribution function (ODF). Bouza et al. (2021) generalized convolution to manifold-valued convolutions using Volterra Series, preserving its equivariance. With the generalization of convolution to more complex group actions than translation, several authors (Gens and Domingos, 2014; Cohen and Welling, 2016a; Weiler et al., 2018b,a; Worrall et al., 2017; Kondor and Trivedi, 2018; Bekkers et al., 2018; Andrearczyk et al., 2020; Chakraborty et al., 2018a,b, 2020; Graham et al., 2020) explored the group convolution path for Lie groups and the homogeneous spaces of these groups. Knigge et al. (2022) proposed a separable convolution setup on Lie groups. The relation between group actions, principal bundles and related vector bundles, and convolutional architectures is currently explored (Cohen et al., 2019, 2020; Aronsson, 2022). The latter elucidates important relations between differential geometry of bundles and Reproducible Kernel Hilbert Spaces. Links between partial differential equations, symmetries, and GCNN are studied in Smets et al. (2021). A unifying framework for equivariant DL on manifolds, connecting both the bundle and homogeneous space viewpoint, is given in Weiler et al. (2021) through a notion of coordinate indepencent convolutions.

Most CNNs approach for the processing of DWI signals discards its specific structure. For instance, Golkov et al. (2016) built multi-layer perceptrons in *q*-space for kurtosis and NODDI mappings. However, the importance of spherical equivariant or invariant structure has been acknowledged for some years now. The importance of the extraction of rotationally invariant features beyond Fractional Anisotropy (Basser et al., 1994) has been recognized in series of DWI works. For instance, Caruyer and Verma (2015) developed invariant polynomials of spherical harmonic (SH) expansion coefficients and discussed their application in population studies. Schwab et al. (2013) proposed a related construction using eigenvalue decomposition of SH operators. Novikov et al. (2018) and Zucchelli et al. (2020) argued their usefulness for understanding microstructures in relation to DWI.

Chakraborty et al. (2018a) proposed a rotation equivariant construction inspired by Cohen et al. (2018) for disease classification. The same authors (Banerjee et al., 2019) used a *S*^2^×ℝ^+^ CNN using SHORE function representation for classification in Parkinson's Disease. Sedlar et al. (2020) used a spherical U-Net for f-ODF estimation. The same authors (Sedlar et al., 2021) used a spherical CNN for microstructure parameter estimation, using spherical harmonics representations. Müller et al. (2021) proposed a sixth-D, 3D space and *q*-space NNs with roto-translation/rotation equivalence properties, targeted at DWI data. Poulenard et al. (2022) reviewed several implementations of *SE*(3) neural networks and showcased a comparison among these networks. In their work, steerable CNNs generalize better than group CNNs while dealing with inconsistent distributions between training and testing data for 3D images.

While most equivariant methods use spectral representation of groups, we propose an SE(3) network for DWI data that uses *regular representation* of groups such that the whole model is light-weight, and the implementation for convolution is not only direct but also separable, improving efficiency. A similar idea was used in Chen et al. (2021), for 3D point cloud feature extraction, with, however, important architectural differences due to the nature of input data. Both our method and Chen et al. (2021) implemented regular representations of groups in a separable fashion; however, their separable kernels are only over the spatial and rotation interactions while we additionally split the rotation interactions over 2 axes, making use of the factorization of the icosahedron group into 12 × 5 rotations. We do this to further boost efficiency. Furthermore, Chen et al. (2021) include an attention mechanism in the interaction layers, while instead, we use non-linearities between the separate interaction steps. As our operations are strictly local, including attention mechanism would introduce unnecessary computational overhead, whereas in Chen et al. (2021) the attention mechanism could be critical as a selection mechanism among the global interactions between many points within the point cloud. In addition, we compared our method to Müller et al. (2021) which uses steerable filter bases (spectral representation of groups) for the *SE*(3) group. In our experiments, in comparison with Müller et al. (2021), we found out, however, that our direct convolution implementation of *SE*(3) GCNN does not perform inferior to its steerable alternative, and our method is a lot more light-weight.

## 3 Method

The networks we present will be built from the principle of expanding CNNs to groups and their homogeneous spaces, on which they act by extending convolution operations to functions on groups and their homogeneous spaces. For the rotation group *SO*(3) and the sphere *S*^2^ as *SO*(3)-homogeneous space, the common path for implementing convolutions/correlations is to use irreducible representations (Cohen and Welling, 2016b). This approach can be computationally very intensive, unless one restricts to very low-order irreducible representations, with a resolution trade-off worse than the approximation of *SO*(3) by the icosahedral rotation group. So we do not follow that path here.

In the next section, we provide the theoretical background for extending convolutions to functions on groups. For the reader's convenience, standard concepts from group theory and group actions that are used to build our new convolution layers are presented in Appendix 1.

### 3.1 Generalized convolution operations

Classical CNNs use the standard convolution operation on ℝ^*n*^: for *h*, κ:ℝ^*n*^ → ℝ, where *h* is the signal and κ is the kernel, the operation is defined as


(1) $$\begin{array}{l} h*\kappa \left(\text{x}\right)=\int_{{\mathbb{R}}^{n}}h\left(\text{y}\right)\kappa \left(\text{x}-\text{y}\right)d\text{y},\;\text{x}=\left(x_{1},\ldots ,x_{n}\right),y=\left(y_{1},\ldots y_{n}\right). \end{array}$$

Here ℝ^*n*^ is the underlying space of the function, *n* = 2 for 2D images, and *n* = 3 for 3D volumetric images. This operation can be extended to vector-valued functions (i.e., functions from ℝ^*n*^ → ℝ^*m*^, they have *m* *channels*) and multiple kernels, and this is of course at the heart of the definition of a convolutional layer in a CNN.

Rewrite Equation 1 as


(2) $$\begin{array}{l} h*\kappa \left(\text{x}\right)=\int_{T^{n}}h\left(\text{y}\right)L_{\text{y}}\kappa \left(\text{x}\right)d\text{y},\;L_{\text{y}}\kappa :\text{x}\mapsto \kappa \left(\text{x}-\text{y}\right). \end{array}$$

*L*_*y*_κ *translates* the kernel κ by vector *y*. This is the *left regular representation* of 𝕋^*n*^ on the space of kernels (see Appendix 1.2.5). Using the regular representation, one gets that the standard convolution (Equation 1) is *translation-equivariant*, a property generally acknowledged as the main source of success for CNNs:


(3) $$\begin{array}{l} \left(L_{\text{z}}h\right)*\kappa \left(\text{x}\right)=L_{\text{z}}\left(h*\kappa \right)\left(\text{x}\right)=h*\kappa \left(\text{x}-\text{z}\right). \end{array}$$

In Appendix 1.2.5, a general definition for the regular representation is given for a Lie group *G* acting on a homogeneous space ℳ. It is defined by $\left(L_{g}f\right)\left(m\right)=f\left(g^{-1}m\right)$. This is in particular the case when ℳ is a principal homogeneous space of *G*, and especially when ℳ = *G*. This leads to a generalization of convolutions for functions defined on a group *G*: if *h*, κ:*G* → ℝ, h*_G_κ, or simply h*κ, if there is no ambiguity, is defined by


(4) $$\begin{array}{l} h*\kappa \left(g\right)=\int_{G}h\left(u\right)L_{u}\kappa \left(g\right)du=\int_{G}h\left(u\right)\kappa \left(u^{-1}g\right)du. \end{array}$$

Here, *du* refers to a Haar measure in *G* (Diestel and Spalsbury, 2014). This operation is equivariant to transformations in the group with respect to the regular representation essentially exactly as in Equation 3:


(5) $$\begin{array}{l} \forall v\in G,\;\left(L_{v}h\right)*\kappa \left(g\right)=L_{v}\left(h*\kappa \right)\left(g\right)=h*\kappa \left(v^{-1}g\right). \end{array}$$

This operation is also equivariant for the left-representation of G.

We deal rarely directly with functions whose domain is a non-trivial group, such as *SE*(3), or data “indexed” by a non-trivial group. The domain is instead a homogeneous space of the group of interest, such as ℝ^3^ or the sphere *S*^2^ for the groups in this work. In that situation, kernel convolution generalizes to a *lifting* operation that produces a new function, this time defined on the group. If *f*:ℳ → ℝ and *k*:ℳ → ℝ are the function and the kernel, respectively, define f*_lifting_*k*, or just f*k, if there is no ambiguity, by


(6) $$\begin{array}{l} f*k\left(g\right)=\int_{ℳ}f\left(m\right)k\left(g^{-1}m\right)dm=\int_{ℳ}f\left(m\right)L_{g}\kappa \left(m\right)dm. \end{array}$$

and this convolution operation is equivariant with respect to actions in *G*:


(7) $$\begin{array}{l} \forall v\in G,\left(L_{v}f\right)*k\left(g\right)=L_{v}\left(f*k\right)\left(g\right)=f*k\left(h^{-1}g\right). \end{array}$$

A bit of caution here, as the first regular representation acts on a function *f*:ℳ → ℝ while the second acts on the function *f*:*G* → ℝ. Once the function is lifted onto this group *G*, *group convolutions* on *G* can be performed on the lifted signals as in Equation 4,

The group convolutions and lifting can be stacked in layers like a standard CNN, and this stacking preserves equivariance, producing equivariant layers. Features at the last group convolution layer can be *projected* back onto the original space of the function by summarizing feature responses over the group. It is similar to max-pooling-like operations in a standard CNN. This type of operation will provide invariance.

Therefore, a roadmap for *group convolutions* can be summarized as follows:

- Lifting the function signals to the desired group.
- Group convolutions on the lifted signals.
- Projecting the signals back onto the original space.

We formulate these operations in the following sections.

#### 3.1.1 Lifting layer

A function $f:ℳ\to {\mathbb{R}}^{N_{0}}$ can be *lifted* to the group *G* via a kernel $\kappa :ℳ\to {\mathbb{R}}^{N_{1}}$ by


(8) $$\begin{array}{l} f*\kappa \left(g\right)={\left(\overset{N_{0}}{\underset{j=1}{\sum }}\int_{ℳ}f_{j}\left(m\right)\kappa_{i}\left(g^{-1}m\right)dm\right)}_{i=1}^{N_{1}} \end{array}$$

This is a direct extension of Equation 6 to vector-valued functions *f*. *N*_0_ is the number of input channels, and *N*_1_ the number of output channels. In practice, in this work, the input function is scalar-valued, i.e., *N*_0_ = 1.

#### 3.1.2 Group convolution layer

A feature function $F:G\to {\mathbb{R}}^{N_{l}}$ is transformed by a convolution kernel $K:G\to {\mathbb{R}}^{N_{l+1}}$ by


(9) $$\begin{array}{l} F*K\left(g\right)={\left(\overset{N_{l}}{\underset{j=1}{\sum }}\int_{G}F_{j}\left(h\right)K_{i}\left(h^{-1}g\right)dh\right)}_{i=1}^{N_{l+1}}. \end{array}$$

Here *N*_*l*_ is the number of channels from the output of the last layer (equivalent to the number of input channels for the current layer), and *N*_*l*+1_ is the number of output channels for the current layer.

#### 3.1.3 Projection layer

If needed, feature map *F*:*G* → ℝ^*n*^ can be projected to a function *f*:ℳ → ℝ^*n*^ by summarizing on the fibers (see appendix 1.2.3.)


(10) $$\begin{array}{l} \bar{F}\left(m\right)=\underset{h\in G_{m_{0}}}{\max}F\left(gh\right),\;\text{for any }g\text{ with }g.m_{0}=m, \end{array}$$

where the max is computed component-wise. This operation is equivariant: $\bar{L_{k}F}=L_{k}\bar{F}$.

#### 3.1.4 Activation functions and separable kernels

A point-wise activation function α, such as ReLU, is trivially equivariant *L*_*g*_(α*f*) = α(*L*_*g*_*f*). On manifolds with an underlying product structure, ℳ = ℳ_1_×ℳ_2_ - this includes homogeneous spaces and groups - one can choose separable kernels κ = κ_ℳ_1__⊗κ_ℳ_2__, and activation functions can be intertwined in between Equations 8, 9. For instance, lifting Equation 8 can be replaced by


(11) $$\begin{array}{r} f{*}^{\alpha }\kappa \left(g\right)=\overset{K}{\underset{i=1}{\sum }}\int_{{ℳ}_{1}}\\ \alpha \left(\int_{{ℳ}_{2}}f\left(m_{1},m_{2}\right)\kappa_{2}\left(g^{-1}m_{2}\right)dm_{2}\right)\kappa_{1}\left(g^{-1}m_{1}\right)dm_{1}, \end{array}$$

where *^α^κ is a shortcut notation for the intertwining of the kernel and activation function. It is easily seen that it preserves equivariance. Having separable kernels increases the efficiency of the model since it increases weight sharing. For example, instead of having kernels defined in ℝ^3^×*S*^2^, we have kernels defined in ℝ^3^ and in *S*^2^. In this way, all voxels in ℝ^3^ share the same spherical kernels. This is used in this work.

The spaces used in this work are ℝ^3^, the sphere *S*^2^, and the product space ℝ^3^×*S*^2^. The groups that we consider are the group of translations of ℝ^3^, 𝕋^3^ ≃ ℝ^3^, the group *SO*(3) or 3D rotations, the direct product 𝒢 = 𝕋^3^ × *SO*(3), and the special Euclidean group *SE*(3) = *SO*(3)⋉𝕋^3^. Note that though 𝒢 and *SE*(3) are isomorphic as manifolds, they are not as groups: in 𝒢, $\left(\vec{t},R\right).\left(\vec{s},S\right)=\left(\vec{t}+\vec{s},RS\right)$ while in *SE*(3), $\left(R,\vec{t}\right).\left(S,\vec{s}\right)=\left(RS,\vec{t}+R\vec{s}\right)$. This is also reflected in their respective actions in Table 1, which shows the different combinations of spaces and groups. We refer the readers to Gerken et al. (2023) for more detailed theoretical foundation.

**Table 1:** Groups and homogeneous spaces in this work.

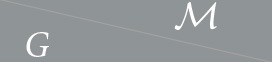	**ℝ^3^, *x***	** $S^{2},\vec{v}$ **	** ${\mathbb{R}}^{3}\times S^{2},\left(x,\vec{v}\right)$ **
$T^{3},\vec{t}$	$x+\vec{t}$		
*SO*(3), *R*		$R\vec{v}$	
$T^{3}\times SO\left(3\right),\left(\vec{t},R\right)$	$x+\vec{t}$	$R\vec{v}$	$\left(x+\vec{t},R\vec{v}\right)$
$SE\left(3\right),\left(R,\vec{t}\right)$	$Rx+\vec{t}$	$R\vec{v}$	$\left(Rx+\vec{t},R\vec{v}\right)$

For each group and each homogeneous space, typical elements are provided, as well as the action of the group element on the space element. Entries left empty are not used or fail to be homogeneous spaces for standard group actions on them.

### 3.2 Discretization of spherical signals

The way spherical signals are numerically handled have major implications for our networks. A DWI signal is treated as a discretization of a signal *f*:ℝ^3^×*S*^2^ → ℝ. DWIs are acquired, for each voxel, at *N* fixed directions *p*_1_, …, *p*_*N*_ on *S*^2^ (here *N* = 90). These are represented in two different ways.

- Type 1. Ignoring the spherical structure, at each voxel *x*, we get a measurement vector
  $f\left(x\right)=\left(f\left(x,p_{1}\right)\ldots ,f\left(x,p_{N}\right)\right)\in {\mathbb{R}}^{N}$. Thus an image is a mapping *I*:ℝ^3^ → ℝ^*N*^.
- Type 2. A signal at voxel *x* is interpolated as a proper spherical function $f\left(x,\vec{v}\right)=W\left(v;v_{1},\ldots ,v_{N}\right)$ where *W* is a Watson kernel (Jupp and Mardia, 1989). An image from this type is a mapping *I*:ℝ^3^×*S*^2^ → ℝ.

### 3.3 Direct convolution and discretization of groups

**Figure 1:**
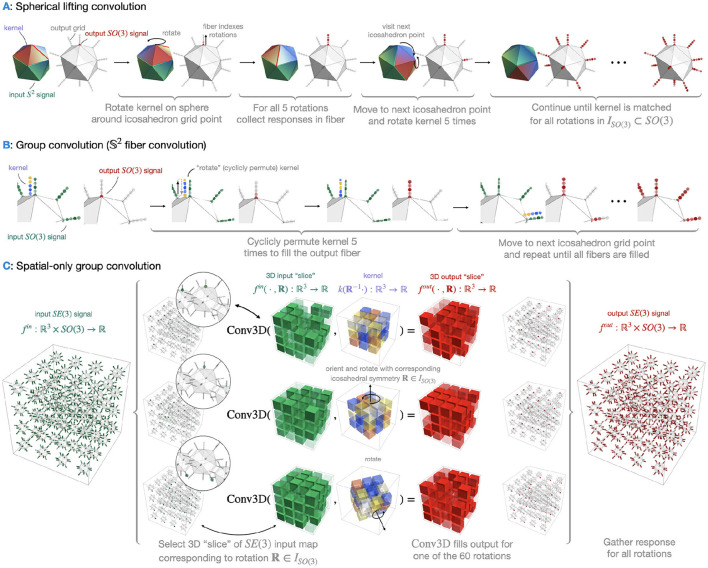
Three group convolution operators used in this paper. **(A)** shows the spherical part of the separable lifting convolution. The star-shaped kernel translates (in this case translation is equivalent to rotation) to the 12 icosahedron vertices like a spider crawling on a sphere. At each vertex location, the kernel rotates five times aligned with the edges of the icosahedron and gets five responses from all the orientations. Therefore, at each vertex, the output is a fiber consisting of five elements. There are in total 60 responses from all 12 vertices, and thus 60 rotation matrices to translate the kernel, assembling a discretization of *SO*(3) - *I*_*SO*(3)_. **(B)** shows the spherical part of the separable group convolution. The kernel is then defined at each fiber and is rotated (permuted) again for five times to get the responses of different orientations, as in the lifting convolution. **(C)** shows the spatial part of the separable convolution (the spatial convolution is the same in the lifting and group convolution; thus, we only show one). The spatial kernel is a 3D grid. The grid is rotated to convolve with all 60 spherical responses. The kernel is rotated 60 times, using the same icosahedral symmetry rotations as those on which the input is sampled.

Unlike existing methods that use generalized Fourier-type transforms to perform convolution on spheres (Cohen et al., 2018; Gens and Domingos, 2014; Cohen and Welling, 2016a; Weiler et al., 2018a; Worrall et al., 2017; Kondor and Trivedi, 2018; Bekkers et al., 2018; Andrearczyk et al., 2020; Chakraborty et al., 2018a,b, 2020), we implement the convolution for spheres directly as in classical 2D CNNs in the image analysis field. We first discretize the sphere *S*^2^ using an icosahedron. To lift the function from the sphere to the *SO*(3) group, we define a star-shaped kernel *k*:*S*^2^↦ℝ with a limited support. The kernel then moves around the discretized sphere and convolves with signals at each vertex of the icosahedron. It rotates five times at each icosahedral vertex according to the fives edges each vertex has, and collects convolutional responses from all five rotations. In this way, the spherical function is lifted to *SO*(3), which is discretized by *I*_*SO*(3)_—the 60 rotational symmetries of an icosahedron. This corresponds to Equation 8 and is shown in Figure 1A. For the *SO*(3) group convolution layer, the kernel is defined on *SO*(3), which is represented by the icosahedral symmetries. Here, we specially design the kernel in the way that the support of it covers exactly a fiber. Therefore, we rotate (permute) the kernel at each fiber, convolve the rotated kernels with the fiber, and move the kernel to the next fiber. This is how Equation 9 is implemented, and more details can be found in Figure 1B. With the impact of discretization of the groups and the interpolation of signals, we lose the benefits of learning from raw data. However, the experiments show that the 60 icosahedral symmetries can approximate the *SO*(3) group well enough such that the models can deal with rotational variations in the data that are different from the rotations used in the discretization.

### 3.4 Generic networks used in this work

We present four constructions in which gradual levels of complexity in group actions are introduced. This can be seen as a group action ablation study. The precise description of each network will be provided in Section 4.

#### 3.4.1 Group of translations 𝕋^3^

**Figure 2:**
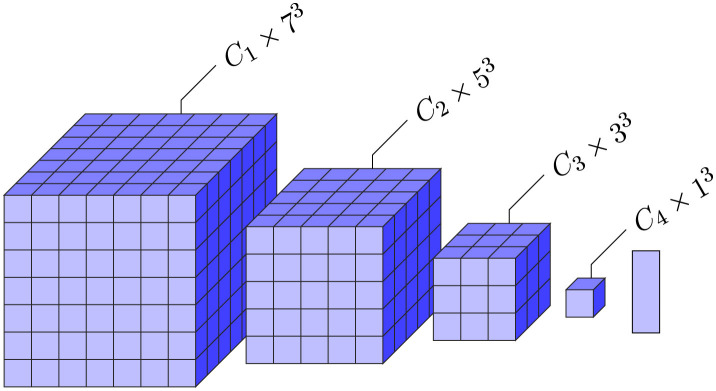
Illustration of the classical CNN. In the grids shown above, which assembles the dimensions of feature maps in the later experiments. Each voxel in the *ith* layer contains *C*_*i*_ values, indicating the numbers of channels. *C*_1_ here is the number of signal values each voxel from the original scan, thus 90. Due to striding, the grid shrinks to 1 voxel after 3 convolutional layers and then is fed into a fully connected layer for classification.

The *S*^2^-structure of the signal is ignored, using the Type 1 discretization. The group being 𝕋^3^, just another name for ℝ^3^, we just obtain a standard CNN, ignoring rotational information. An illustration can be found in Figure 2.

#### 3.4.2 *SO*(3)

This time the spatial structure is ignored, and each voxel provides a spherical data point. Type 2 discretization is used. The GCNN takes as input a spherical function and will classify it by performing *SO*(3)-lifting, *SO*(3)-convolutions and summarization. The convolved function on *SO*(3) is then projected back to *S*^2^ by this summarization. It is illustrated in Figures 1A, B. This model is a fully equivariant implementation of *SO*(3) group convolution followed by the work in Liu et al. (2021), which does not hold global equivariance.

#### 3.4.3 𝕋^3^ × *SO*(3)

Spatial and spherical structures are decoupled. This implies a standard spatial CNN dealing with only voxel translations, and a *SO*(3)-GCNN part for the directional signal. Type 2 discretization is used for spherical signals. The decoupled ℝ^3^-layer and *S*^2^-layer are with group actions 𝕋^3^ and *SO*(3), respectively. The illustration for the *S*^2^-layer can be found in Figures 1A, B, and the illustration for the ℝ^3^-layer can be regarded as only one Conv3D operation in Figure 1C without the rotations. Note that since the spatial convolution does not incorporate rotational equivariance, it does not reflect equivariance of the DWI measurements. I.e., one can expect that when the brain rotates, the spatial patterns rotate, as well as their spherical diffusion signals. This model takes rotation into account in the spherical part of the signal but not the spatial part. The projection at the end collapses the function in the group back to ℝ^3^ by summarizing—in this case, maximizing—over *SO*(3), and the resulting feature map is fed into a fully connected layer to perform the classification task.

#### 3.4.4 *SE*(3)

Type 2 discretization is used, and the network uses the full interplay between spatial roto-translations and corresponding rotations of the spherical signal and is thus fully equivariant to *SE*(3) transformations on the DWI data. Figures 1A, B shows the kernels of the *S*^2^-layer. When the kernel moves from one vertex to another, it follows a specific rotation that maps the one-ring neighborhood of the source vertex to the one-ring neighborhood of the target vertex. At each vertex, the kernel has an *SO*(2) symmetry group structure discretized by 5 rotations. Figure 1C shows the kernel for the ℝ^3^-layer. It is rotated with the same rotation matrices that moved the *S*^2^-kernel as in Figures 1A, B. Since the spatial kernels are cube-shaped grids, interpolation is required while rotating them. Here, we use linear interpolation, which can be easily implemented. To perform the segmentation task, the projection layer collapses the function on *SE*(3) back to ℝ^3^ by summarizing—again, maximizing—over *SO*(3).

## 4 Experiments and results

**Figure 3:**
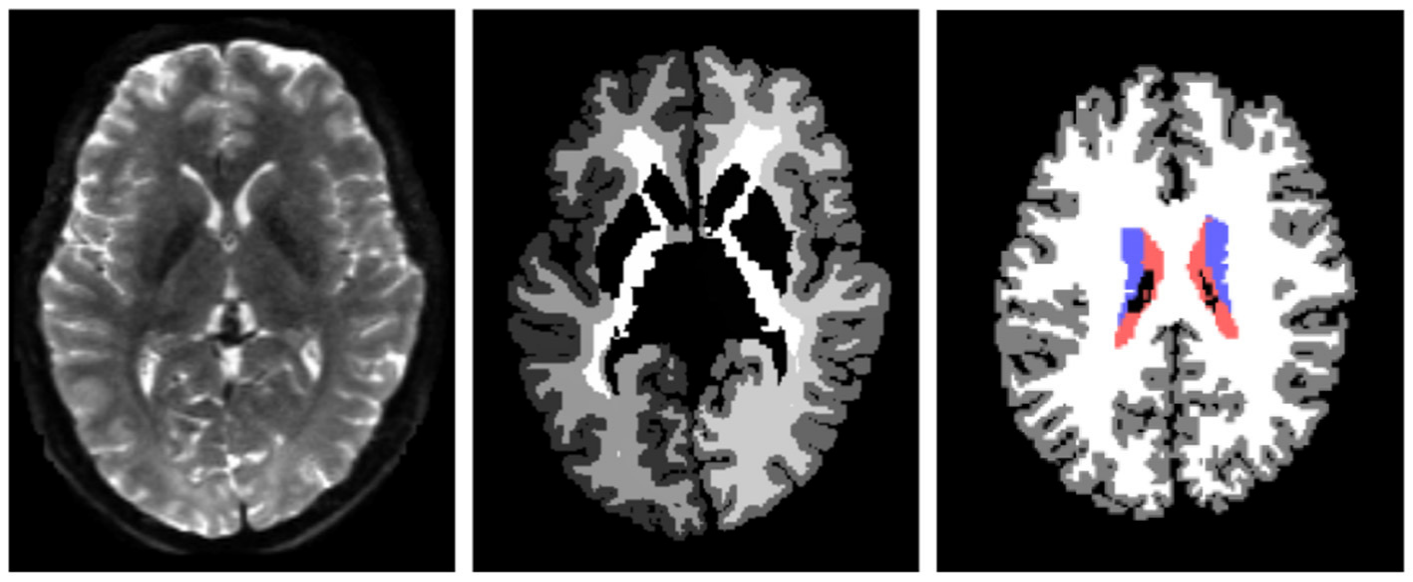
Left to right: original diffusion data, the ground truth segmentation, and the processed ground-truth that we are going to learn from. The label colors for CSF, subcortical, white matter, and gray matter are red, blue, white, and gray, respectively. The figures only illustrate the data, and they are not necessarily from the same slice of the same scan.

In this section, we first list all the detailed network setups, after which we present the results of the experiments. We evaluate our method on the DWI brain dataset from the human connectome project (HCP) (Van Essen et al., 2013). We classify the human brains into four regions - cerebrospinal fluid (CSF), subcortical, white matter (WM), and gray matter (GM). An illustration of the task can be found in Figure 3.

We use the preprocessed DWI data (Van Essen et al., 2013) and normalize each DWI scan for the *b*-1000 images with the voxel-wise average of the *b*_0_. We use the brain masks provided in the dataset to obtain the voxels of interest, while background is ignored. The labels provided with the T1-image are transformed to the DWI using nearest neighbor interpolation (Figure 3). The resolution of the DWI images is 145 × 174 × 145, and the resolution of the T1-images is 260 × 311 × 260. Focal Loss (Lin et al., 2018) is used to counter the class imbalance of the four brain regions. For Focal Loss, all experiments use γ = 2 and use α = (0.35, 0.35, 0.15, 0.15) for CSF, subcortical, WM, and GM, respectively. For the Watson Kernel, all experiments that used this interpolation (Type 2 discretization) have κ = 10. Batch size for all experiments is 100, and the learning rate for all experiments is 0.001.

### 4.1 Experimental setup

Since each DWI scan is highly resoluted, it is not feasible to use a whole image as input to the networks. Therefore, to reduce the computational burden, as inputting a full DWI volume is intractable, we use spatial windows of *N*^3^ voxels, with *N* = 1 for the *SO*(3)-action network and *N* = 7 for the rest. In addition, due to the effect of striding in spatial convolution, the 7^3^ grid of voxels shrinks to 1^3^ after 3 spatial convolutions. Therefore, a separable convolution layer (for both 𝕋^3^ × *SO*(3) and *SE*(3) actions) is equivalent to a single *SO*(3) convolution layer when the grid shrinks to 1^3^ since the spatial convolution becomes trivial. *S*^2^ is discretized by a regular icosahedron. *SO*(3) is discretized as the icosahedral rotation group with 60 elements. Each vertex of the icosahedron is fixed by five rotations, isomorphic to the subgroup of *SO*(2) consisting of rotations of angle 2*kπ*/5, *k* = 0…4. This is, of course, the discretization used for *SO*(2).

To validate the proposed *SE*(3) network, we first provide an ablation study of our proposed four types of networks based on different group actions. Then, we compare it with Müller et al. (2021), which implements an *SE*(3)-GCNN using *irreducible representations*.

For the ablation study, based on the networks that were introduced above and in alignment with the networks presented in Liu et al. (2022), we design our experiments for them. For each experiment, in order to explore the impact of model capacity on the performance, we construct two models with high and low capacities, respectively, denoted by the superscription + and -. We choose the architectures for the models with low capacity by trying out different complexities and depths and picking the one with the lowest capacity with the same level of performance. Then for the models with high capacity, we simply increase the numbers of kernels in each layer of the models with low capacity.

Detailed descriptions of all the experiments are reported below, and a summary of the experiments can be found in Table 2.

**Table 2:** Criteria and properties of experiments.

**Experiment**	***G***	**#Params**	**#Epochs**
*I*:ℝ^3^ → ℝ^*N*^			
Classical^-^	𝕋^3^	13,539	34
ClassicalAug^-^			66
Classical^+^		972,694	19
ClassicalAug^+^			67
*I*:ℝ^3^×*S*^2^ → ℝ			
Baseline^-^	*SO*(3)	286	31
BaselineAug^-^			45
Baseline^+^		2,104	31
BaselineAug^+^			54
OursDecoupled^-^	𝕋^3^×*SO*(3)	2,514	41
OursDecoupledAug^-^			80
OursDecoupled^+^		59,914	15
OursDecoupledAug^+^			54
OursPart^-^	*SE*(3)*	2,514	41
OursPartAug^-^			49
OursPart^+^		59,914	15
OursPartAug^+^			48
OursFull^-^	*SE*(3)	2,514	41
OursFullAug^-^			86
OursFull^+^		59,914	15
OursFullAug^+^			42

*SE*(3)* indicates the rotations in the spatial part are only a part of the rotations used in the spherical part.

### 4.2 Ablation study

#### 4.2.1 𝕋^3^-Classical CNN

The architecture we use is *ReLU*(ℝ^3^ conv)−*ReLU*(ℝ^3^conv)−*ReLU*(ℝ^3^conv)−FC with network setups of a low capacity and a high capacity. FC here is a fully connected layer. We label the small network (90 − 5−5 − 5−4) Classical^-^ and the big network (90 − 120 − 120 − 90 − 4) Classical^+^.

#### 4.2.2 *SO*(3)-Baseline

In the experiments, we use the *ReLU*(lift) −*ReLU*(gconv)−project−FC architecture as was used in Liu et al. (2021) but with true *SO*(3)-convolution. The projection layer takes the maximum of the five rotations to collapse the function back to the sphere. We experimented various sizes of the network (10 − 20−*proj*.−4 and 20 − 40−*proj*.−4), in addition to the setup used in Liu et al. (2021) (1 − 5−*proj*.−4). The network that has the biggest size did not seem to improve the second biggest one; thus, we omit it in this paper. Based on the size of the experiments, we call the small network Baseline^-^ and the big network Baseline^+^.

#### 4.2.3 𝕋^3^ × *SO*(3)-OursDecoupled

**Figure 4:**
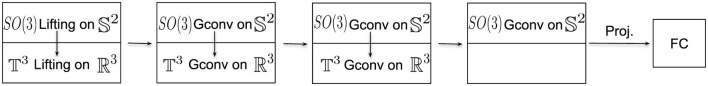
Architecture of the network with group action 𝕋^3^ × *SO*(3). Each block is a convolutional layer split into two separable layers. The vertical arrows in each block show the separable convolutions. First, the spherical convolution is applied, followed by the spatial convolution. The last block before the FC layer is equivalent to a single *S*^2^-layer as explained in Section 4.1. Illustrations of ReLU actions are omitted for visualization simplicity.

We use the architecture *ReLU*(lift)−*ReLU*(gconv)−*ReLU*(gconv)−*ReLU*(gconv) −project−FC. Using separability discussed in Section 3.1.4, a convolution layer (including lifting) is split into two, and ReLU activation is added between separable layers as well. An illustration of the architecture can be found in Figure 4.

We again experiment with two sizes of the network - a small one and a big one. The small network has 5 − 5−5 − 5−5 − 5−5−*proj*.−4 kernels for each layer, while the big network has 10 − 20 − 20 − 40 − 40 − 20 − 10−*proj*.−4. We label them OursDecoupled^-^ and OursDecoupled^+^.

#### 4.2.4 *SE*(3)-ours

**Figure 5:**
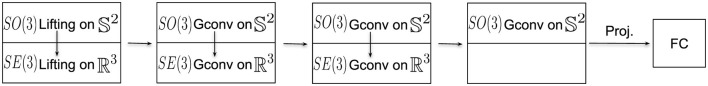
Architecture of the network with group action *SE*(3).

Here too we use the separable setup described in Section 3.1.4. Thus, a layer is again split into two layers - an *S*^2^-layer and an ℝ^3^-layer, both for lifting and group convolution. The *S*^2^-layer is defined as shown in Figures 1A, B. We rotate the ℝ^3^ kernels and the *S*^2^ kernels using the same actions. The rotational actions of the kernels can be represented by 60 rotation matrices and is equivalent to the discretization of the *SO*(3) rotation group using the icosahedral symmetry group, as shown in Figure 1C. As in Section 4.2.3, we use the *ReLU*(lift)−*ReLU*(gconv) −*ReLU*(gconv)−*ReLU*(gconv)−project−FC architecture. After the separation of the layers, the illustration is showcased in Figure 5. As in Section 4.2.3, ReLU activations are added between separable layers as well.

In addition, we intend to explore the impact of the equivariance we imposed in ℝ^3^ in this section. As was explained above, we align the rotations of the ℝ^3^ kernel with the ways the *S*^2^ kernel moved on the sphere, which is discretized by the 60 rotation symmetries of an icosahedron. At a vertex *x*_*i*_, *i*∈1, …, 12 of an icosahedron, there exists a stabilizer *SO*(3)_*x*_*i*__ discretized by 5 equally divided rotations that keep *x*_*i*_ unchanged. Therefore, we also experiment a partial equivariance in the ℝ^3^ roto-translational convolution. This means at each vertex *x*_*i*_ of the icosahedron, we only take 1 out of the 5 rotations that discretized *SO*(3)_*x*_*i*__ instead of using all of them to rotate the spatial kernel. Note that the partially equivariant models are only fully *SE*(3)-equivariant when the kernels have a subgroup *SO*(2) symmetry in them (Bekkers, 2019; Thm 1), which we do not impose and thus equivariance is not guaranteed.

Again, we experiment with two sizes of the network with 5 − 5−5 − 5−5 − 5−5−*proj*.−4 and 10 − 20 − 20 − 40 − 40 − 20 − 10−*proj*.−4 kernels, respectively. Therefore, we generate four experiments for this section: OursFull^-^, OursPart^-^, OursFull^+^, and OursPart^+^.

#### 4.2.5 Data augmentation experiments

To validate the robustness of GCNNs against data variation modeled by group actions, we train all the proposed models with augmented data as well. Each data sample (grid of 7^3^ or 1^3^) is randomly rotated on the fly before being fed into the model. To prevent interpolation, the rotations used to transform the data are sampled from a octohedral symmetry group. For DWI data that have directional signals in each voxel, the directions of the signals (*b*-vectors) in each voxel rotate with the voxel grid. In order to guarantee the signal values in each voxel are from the same orientations after augmentation, we interpolate the function values at the orientations-of-interest using the rotated *b*-vectors. Therefore, for Type 1 discretization, we interpolate function values at the original *b*-vectors, and for Type 2 discretization, we interpolate at the pre-defined icosahedron as demonstrated above.

### 4.3 Results

As was done in Liu et al. (2021), we trained all networks using **1** scan, validated using **1** scan, and tested using **50** scans. We evaluate the accuracies and Dice scores of the classification of the four regions, respectively, and the overall classification accuracy across all test scans. We have also tried training models with more scans (5 or 10); it does not seem to improve the results significantly. Therefore, we choose to use 1 scan for training. For each class, the accuracy is calculated by $\frac{\#CorrectPredictions}{\#ClassSamples}$, and the Dice score is calculated by $\frac{2TP}{2TP+FP+FN}$ for the class. The overall accuracy is calculated by $\frac{\#CorrectPredictions}{\#AllSamples}$.

We trained all models until they converge and before overfitting; thus, models of different capacities and different setups are stopped at different epochs. Each model is trained with both original data and augmented data. Details can be found in Table 2.

**Figure 6:**
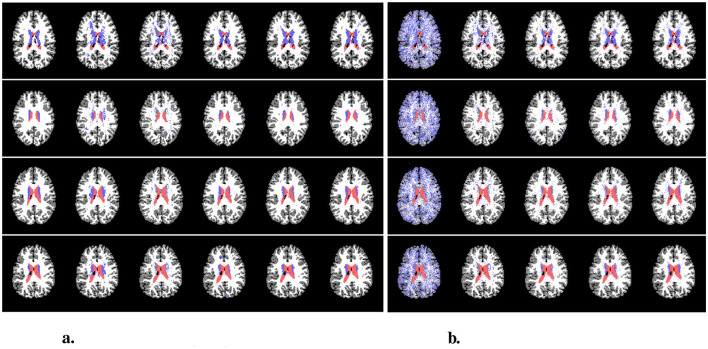
Examples of predictions. **(A)** shows the predictions from the original test set, and **(B)** shows the predictions from the augmented (rotated) test set. In **(A)**, from left to right are ground-truth, Classical^+^, Baseline^+^, OursDecoupled^+^, OursPart^+^, and OursFull^+^. In **(B)**, from left to right are Classical^+^, Baseline^+^, OursDecoupled^+^, OursPart^+^, and OursFull^+^. The colors of CSF, subcortical, WM, and GM are red, blue, white, and gray, respectively. **(A)** Predictions using original data. **(B)** Predictions using rotated data.

The Dice scores and accuracies of models of low capacity can be found in Tables 3, 4, while the Dice scores and accuracies of models of high capacity can be found in Tables 5, 6. The numbers shown in all the tables are the average value and standard deviation across 50 test scans. Examples of predictions compared with the ground truth can be found in Figure 6A.

**Table 3:** Statistics of dice scores from experiments using models of low capacity.

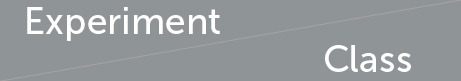	**CSF**	**Subcortical**	**WM**	**GM**
*I*:ℝ^3^ → ℝ^*N*^				
Classical^-^	0.756 ± 0.07	0.376 ± 0.043	0.834 ± 0.011	0.839 ± 0.02
ClassicalAug^-^	0.625 ± 0.11	0.128 ± 0.021	0.77 ± 0.017	0.806 ± 0.017
*I*:ℝ^3^×*S*^2^ → ℝ				
Baseline^-^	0.75 ± 0.073	0.185 ± 0.04	0.801 ± 0.012	0.83 ± 0.011
BaselineAug^-^	0.741 ± 0.074	0.232 ± 0.048	0.805 ± 0.014	0.835 ± 0.011
OursDecoupled^-^	**0.817**±0.051	**0.705**±0.033	**0.867**±0.009	**0.909**±0.007
OursDecoupledAug^-^	0.775 ± 0.063	0.639 ± 0.038	0.851 ± 0.01	0.886 ± 0.009
OursPart^-^	0.807 ± 0.048	0.658 ± 0.037	0.865 ± 0.009	0.899 ± 0.008
OursPartAug^-^	0.78 ± 0.06	0.643 ± 0.037	0.849 ± 0.01	0.886 ± 0.009
OursFull^-^	0.769 ± 0.06	0.621 ± 0.038	0.854 ± 0.01	0.891 ± 0.008
OursFullAug^-^	0.772 ± 0.061	0.637 ± 0.037	0.846 ± 0.01	0.884 ± 0.009

The bold values highlight the maximum value in the column.

**Table 4:** Statistics of classification accuracy from all experiments using models of low capacity.

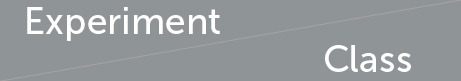	**CSF**	**Subcortical**	**WM**	**GM**	**Overall**
*I*:ℝ^3^ → ℝ^*N*^					
Classical^-^	0.792 ± 0.08	0.415 ± 0.053	**0.879**±0.024	0.789 ± 0.034	0.806 ± 0.017
ClassicalAug^-^	0.662 ± 0.105	0.088 ± 0.017	0.808 ± 0.042	0.801 ± 0.039	0.761 ± 0.014
*I*:ℝ^3^×*S*^2^ → ℝ					
Baseline^-^	0.742 ± 0.082	0.145 ± 0.04	0.804 ± 0.024	0.85 ± 0.016	0.788 ± 0.011
BaselineAug^-^	0.785 ± 0.074	0.202 ± 0.055	0.793 ± 0.028	0.858 ± 0.018	0.791 ± 0.012
OursDecoupled^-^	**0.844**±0.061	0.741 ± 0.033	0.833 ± 0.02	**0.934**±0.013	**0.878**±0.009
OursDecoupledAug^-^	0.769 ± 0.087	0.716 ± 0.04	0.854 ± 0.023	0.87 ± 0.023	0.853 ± 0.01
OursPart^-^	0.787 ± 0.068	0.717 ± 0.032	0.848 ± 0.019	0.906 ± 0.016	0.868 ± 0.009
OursPartAug^-^	0.772 ± 0.081	**0.752**±0.036	0.848 ± 0.021	0.87 ± 0.022	0.852 ± 0.01
OursFull^-^	0.81 ± 0.065	0.692 ± 0.029	0.857 ± 0.022	0.874 ± 0.019	0.856 ± 0.01
OursFullAug^-^	0.783 ± 0.077	0.711 ± 0.054	0.855 ± 0.023	0.864 ± 0.021	0.85 ± 0.01

The bold values highlight the maximum value in the column.

**Table 5:** Statistics of dice scores from experiments using models of high capacity.

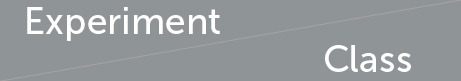	**CSF**	**Subcortical**	**WM**	**GM**
*I*:ℝ^3^ → ℝ^*N*^				
Classical^+^	0.804 ± 0.053	0.583 ± 0.036	0.856 ± 0.011	0.893 ± 0.009
ClassicalAug^+^	0.752 ± 0.069	0.407 ± 0.044	0.828 ± 0.011	0.849 ± 0.017
*I*:ℝ^3^×*S*^2^ → ℝ				
Baseline^+^	0.754 ± 0.069	0.334 ± 0.037	0.805 ± 0.013	0.841 ± 0.012
BaselineAug^+^	0.748 ± 0.072	0.311 ± 0.037	0.796 ± 0.016	0.845 ± 0.011
OursDecoupled^+^	0.827 ± 0.047	0.716 ± 0.044	0.878 ± 0.009	0.903 ± 0.01
OursDecoupledAug^+^	0.79 ± 0.053	0.721 ± 0.033	0.87 ± 0.009	0.902 ± 0.007
OursPart^+^	**0.834**±0.045	**0.752**±0.034	**0.878**±0.009	**0.914**±0.007
OursPartAug^+^	0.789 ± 0.059	0.736 ± 0.035	0.872 ± 0.009	0.902 ± 0.008
OursFull^+^	0.788 ± 0.05	0.746 ± 0.034	0.877 ± 0.008	0.909 ± 0.006
OursFullAug^+^	0.792 ± 0.051	0.737 ± 0.031	0.873 ± 0.009	0.907 ± 0.007

The bold values highlight the maximum value in the column.

**Table 6:** Statistics of classification accuracy from all experiments using models of high capacity.

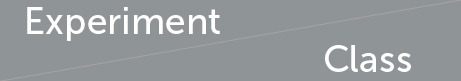	**CSF**	**Subcortical**	**WM**	**GM**	**Overall**
*I*:ℝ^3^ → ℝ^*N*^					
Classical^+^	0.815 ± 0.061	0.702 ± 0.026	0.834 ± 0.022	0.89 ± 0.011	0.854 ± 0.012
ClassicalAug^+^	0.687 ± 0.088	0.42 ± 0.04	0.863 ± 0.031	0.818 ± 0.038	0.812 ± 0.015
*I*:ℝ^3^×*S*^2^ → ℝ					
Baseline^+^	0.778 ± 0.07	0.379 ± 0.065	0.784 ± 0.024	0.848 ± 0.02	0.792 ± 0.013
BaselineAug^+^	0.776 ± 0.076	0.351 ± 0.067	0.749 ± 0.029	0.875 ± 0.017	0.789 ± 0.014
OursDecoupled^+^	0.865 ± 0.061	0.783 ± 0.035	0.867 ± 0.017	0.902 ± 0.019	0.879 ± 0.011
OursDecoupledAug^+^	0.821 ± 0.066	0.759 ± 0.052	0.876 ± 0.02	0.891 ± 0.018	0.876 ± 0.008
OursPart^+^	0.819 ± 0.065	0.816 ± 0.031	0.845 ± 0.019	**0.936**±0.011	**0.888**±0.009
OursPartAug^+^	0.756 ± 0.084	0.816 ± 0.033	**0.876**±0.017	0.888 ± 0.017	0.877 ± 0.009
OursFull^+^	**0.896**±0.042	**0.826**±0.023	0.857 ± 0.017	0.912 ± 0.014	0.883 ± 0.008
OursFullAug^+^	0.864 ± 0.048	0.78 ± 0.031	0.866 ± 0.019	0.905 ± 0.016	0.88 ± 0.008

The bold values highlight the maximum value in the column.

#### 4.3.1 The impact of data augmentation

As we can see from the Tables 3–6, models trained with augmented data do not perform better than their counterparts trained with just original data, if not worse. Unlike 2D image datasets in the computer vision community that have various backgrounds and objects in their images, the HCP dataset is very uniform; thus, the distribution of the original training data is expected to be the same as the test set data. However, after augmentation, the distribution of the training data changed and it differs from the test data. Therefore, in this case, data augmentation does not help any of the models since the augmentation does not represent the diversity in this dataset. One extreme would be Classical^-^ vs. ClassicalAug^-^ that can be found in Tables 3, 4, the augmented data confused the model in terms of the subcortical region - a somewhat mixture of white and gray matter which is challenging for models to distinguish. Therefore, from now on, if not specified, we mainly discuss the models and results trained without data augmentation.

#### 4.3.2 The impact of the ℝ^3^ spatial component

It is easy to observe that the the Baseline experiments perform worst among all. This is an anticipated outcome since it is usually the case that neighboring information is an essential type of local features.

#### 4.3.3 Type 1 discretization vs Type 2 discretization

The classical CNNs use Type 1 discretization, while Type 2 discretization is used for the rest of the models. The classical CNNs do not perform as well as models that take into account the spherical geometry with spatial information but performs better than Baseline. However, Classical^-^ is not much better than Baseline^+^ while having far more parameters to train, and Classical^+^ performs even worse than OursDecoupled^-^, OursPart^-^, or OursFull^-^, which have much less training parameters.

The results of the two extreme cases—Baseline that only takes into account spherical geometry but ignore any spatial information and Classical that only looks into the spatial part and discards spherical geometry—show that the voxel geometry and neighboring voxel correlation can both capture some decent amount of information to deal with the segmentation task, but they both have something that the other one cannot grasp, and combining the spherical geometry and the spatial correlation can boost the performance to a promising extent.

#### 4.3.4 The impact of adding an ℝ^3^ part to baseline

On top of the Baseline, the easiest way to add spatial information to the purely voxel-based framework is what was done in OursDecoupled Section 4.2.3—a GCNN on *S*^2^ to learn the geometric signals in individual signals and a regular classical CNN to take into account the local spatial information. We can see from the results that this setup immediately boosted the performance compared to the Baseline. We can also see that OursDecoupled^+^ performs better than OursDecoupled^-^, for the sake of model capacity.

#### 4.3.5 The argument for OursFull not performing the best

For models of low capacity, however, we can observe from Tables 3, 4 that our proposed method performs worse than OursDecoupled^-^. In addition, for models of high capacity, even though we can see that OursFull^+^ and OursPart^+^ improve from their low capacity counterparts more than OursDecoupled^+^, OursFull^+^ does not perform as well as OursPart^+^ as shown in Tables 5, 6. This differs from our expectation since models with full roto-translational equivariance should be more capable of handling variances in data, thus should have better performance. Recall that the HCP dataset (Van Essen et al., 2013) contains scans that are preprocessed and aligned with axes, thus there is little variance in rotation. In this case, enforcing *SE*(3) equivariance in the model can be futile and be even confusing for the model.

To verify this theory, we evaluated all models on the rotated test set. Taking the *N*^3^ (*N* = 1 for Baseline models and *N* = 7 for the rest) grids of voxels we extracted from the test scans, we randomly rotate each grid using a rotation sampled from the octahedral symmetry group to create a new rotated test set. In this way, we do not need to interpolate while rotating, and the rotations are not aligned with the ones we used in our models to rotate the kernels while still resemble a discretization of the *SO*(3) group. Hence, we have two categories of models as well as two categories of the test set: models trained with original data vs. models trained with augmented data, and original test set vs. the randomly rotated test set.

#### 4.3.6 Models trained with data augmentation tested with rotated test set

We see that all models trained with augmented training set have very similar performance results to the same models tested with the original test set, and they all perform better in this task than their counterparts trained with the original training set. This checks with our statement in Section 4.3.1 that the consistency of data distributions of the training and test sets boosts test performance. In this case, we used the same kind of rotations while augmenting the training set and test set; therefore, the consistency of data distributions is maintained. However, this can never be guaranteed in real life. We can see this from Tables 7–10.

**Table 7:** Statistics of dice scores from experiments using rotated data and models of low capacity.

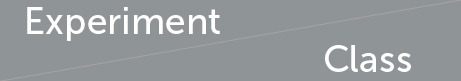	**CSF**	**Subcortical**	**WM**	**GM**
*I*:ℝ^3^ → ℝ^*N*^				
Classical^-^	0.631 ± 0.097	0.101 ± 0.014	0.696 ± 0.019	0.558 ± 0.044
ClassicalAug^-^	0.678 ± 0.094	0.117 ± 0.025	0.775 ± 0.018	0.813 ± 0.019
*I*:ℝ^3^×*S*^2^ → ℝ				
Baseline^-^	0.735 ± 0.076	0.158 ± 0.037	0.799 ± 0.013	0.829 ± 0.011
BaselineAug^-^	0.741 ± 0.074	0.237 ± 0.047	0.804 ± 0.014	0.834 ± 0.011
OursDecoupled^-^	0.708 ± 0.073	0.531 ± 0.033	0.801 ± 0.012	0.851 ± 0.006
OursDecoupledAug^-^	0.771 ± 0.065	0.641 ± 0.036	**0.851**±0.01	0.886 ± 0.009
OursPart^-^	0.714 ± 0.069	0.536 ± 0.035	0.804 ± 0.011	0.851 ± 0.008
OursPartAug^-^	**0.784**±0.059	**0.642**±0.036	0.849 ± 0.01	**0.887**±0.009
OursFull^-^	0.737 ± 0.065	0.517 ± 0.033	0.823 ± 0.01	0.867 ± 0.009
OursFullAug^-^	0.774 ± 0.061	0.636 ± 0.036	0.846 ± 0.01	0.884 ± 0.009

The bold values highlight the maximum value in the column.

#### 4.3.7 Models trained with original data tested with rotated test set

In this section, only models trained without data augmentation are compared and discussed. For models with both low and high capacity, OursFull models have the best performance among other models. OursFull^-^ remains 0.823 accuracy, decreased from 0.856 while OursFull^+^ decreased from 0.883 to 0.84. This is illustrated in Tables 8, 10. In terms of Dice scores, OursFull^-^ performs the best for all classes but the subcortical class, and OursFull^+^ has the best results for **all** classes, as shown in Tables 7, 9.

**Table 8:** Statistics of classification accuracy from experiments using rotated data and models of low capacity.

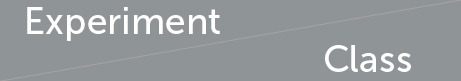	**CSF**	**Subcortical**	**WM**	**GM**	**Overall**
*I*:ℝ^3^ → ℝ^*N*^					
Classical^-^	0.643 ± 0.106	0.24 ± 0.047	0.767 ± 0.051	0.421 ± 0.048	0.563 ± 0.023
ClassicalAug^-^	0.677 ± 0.105	0.08 ± 0.02	0.811 ± 0.044	0.811 ± 0.043	0.767 ± 0.016
*I*:ℝ^3^×*S*^2^ → ℝ					
Baseline^-^	0.733 ± 0.085	0.12 ± 0.035	0.802 ± 0.024	0.852 ± 0.016	0.786 ± 0.011
BaselineAug^-^	0.786 ± 0.074	0.21 ± 0.057	0.793 ± 0.029	0.856 ± 0.018	0.79 ± 0.012
OursDecoupled^-^	0.755 ± 0.076	0.528 ± 0.037	0.779 ± 0.02	**0.871**±0.013	0.81 ± 0.008
OursDecoupledAug^-^	0.765 ± 0.09	0.72 ± 0.038	0.853 ± 0.023	0.871 ± 0.023	**0.853**±0.01
OursPart^-^	0.69 ± 0.084	0.599 ± 0.033	0.791 ± 0.02	0.852 ± 0.018	0.809 ± 0.009
OursPartAug^-^	0.778 ± 0.081	**0.745**±0.038	0.849 ± 0.021	0.87 ± 0.021	0.853 ± 0.01
OursFull^-^	**0.79**±0.067	0.591 ± 0.026	0.835 ± 0.023	0.84 ± 0.022	0.823 ± 0.01
OursFullAug^-^	0.785 ± 0.077	0.707 ± 0.053	**0.854**±0.023	0.865 ± 0.021	0.85 ± 0.01

The bold values highlight the maximum value in the column.

**Table 9:** Statistics of dice scores from experiments using rotated data and models of high capacity.

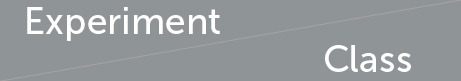	**CSF**	**Subcortical**	**WM**	**GM**
*I*:ℝ^3^ → ℝ^*N*^				
Classical^+^	0.549 ± 0.106	0.124 ± 0.007	0.535 ± 0.014	0.59 ± 0.022
ClassicalAug^+^	0.768 ± 0.066	0.445 ± 0.038	0.82 ± 0.015	0.857 ± 0.014
*I*:ℝ^3^×*S*^2^ → ℝ				
Baseline^+^	0.733 ± 0.076	0.282 ± 0.036	0.799 ± 0.013	0.839 ± 0.012
BaselineAug^+^	0.748 ± 0.072	0.311 ± 0.037	0.796 ± 0.016	0.844 ± 0.011
OursDecoupled^+^	0.702 ± 0.075	0.497 ± 0.037	0.8 ± 0.011	0.829 ± 0.009
OursDecoupledAug^+^	**0.794**±0.054	0.723 ± 0.033	0.87 ± 0.009	0.902 ± 0.007
OursPart^+^	0.734 ± 0.063	0.58 ± 0.033	0.806 ± 0.011	0.862 ± 0.006
OursPartAug^+^	0.791 ± 0.058	**0.736**±0.034	0.872 ± 0.009	0.901 ± 0.008
OursFull^+^	0.74 ± 0.06	0.604 ± 0.034	0.835 ± 0.01	0.877 ± 0.008
OursFullAug^+^	0.79 ± 0.051	0.735 ± 0.03	**0.872**±0.009	**0.907**±0.007

The bold values highlight the maximum value in the column.

**Table 10:** Statistics of classification accuracy from experiments using rotated data and models of high capacity.

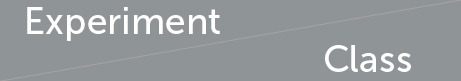	**CSF**	**Subcortical**	**WM**	**GM**	**Overall**
*I*:ℝ^3^ → ℝ^*N*^					
Classical^+^	0.632 ± 0.097	0.452 ± 0.02	0.434 ± 0.018	0.5 ± 0.03	0.471 ± 0.015
ClassicalAug^+^	0.71 ± 0.088	0.517 ± 0.033	0.811 ± 0.038	0.85 ± 0.034	0.812 ± 0.015
*I*:ℝ^3^×*S*^2^ → ℝ					
Baseline^+^	0.769 ± 0.074	0.307 ± 0.059	0.782 ± 0.024	0.846 ± 0.02	0.786 ± 0.013
BaselineAug^+^	0.776 ± 0.076	0.356 ± 0.068	0.749 ± 0.029	0.873 ± 0.017	0.788 ± 0.014
OursDecoupled^+^	0.756 ± 0.082	0.597 ± 0.034	0.797 ± 0.019	0.81 ± 0.019	0.791 ± 0.01
OursDecoupledAug^+^	0.819 ± 0.067	0.761 ± 0.051	0.876 ± 0.019	0.891 ± 0.018	0.876 ± 0.008
OursPart^+^	0.716 ± 0.078	0.635 ± 0.033	0.78 ± 0.021	0.876 ± 0.012	0.819 ± 0.008
OursPartAug^+^	0.762 ± 0.085	**0.811**±0.032	**0.878**±0.018	0.886 ± 0.017	0.877 ± 0.009
OursFull^+^	**0.88**±0.048	0.659 ± 0.028	0.83 ± 0.019	0.868 ± 0.018	0.84 ± 0.009
OursFullAug^+^	0.862 ± 0.049	0.78 ± 0.031	0.865 ± 0.019	**0.904**±0.016	**0.88**±0.008

The bold values highlight the maximum value in the column.

It is worth noticing that Baseline models almost do not suffer from performance drop while applied with rotated data. It is an *SO*(3)-network that preserves rotational equivariance on *S*^2^. For a single-voxel input, the network is very resistant to variations, but the performance of this model is limited due to the lack of spatial interaction and thus in general worse than models with spatial interplay.

**Figure 7:**
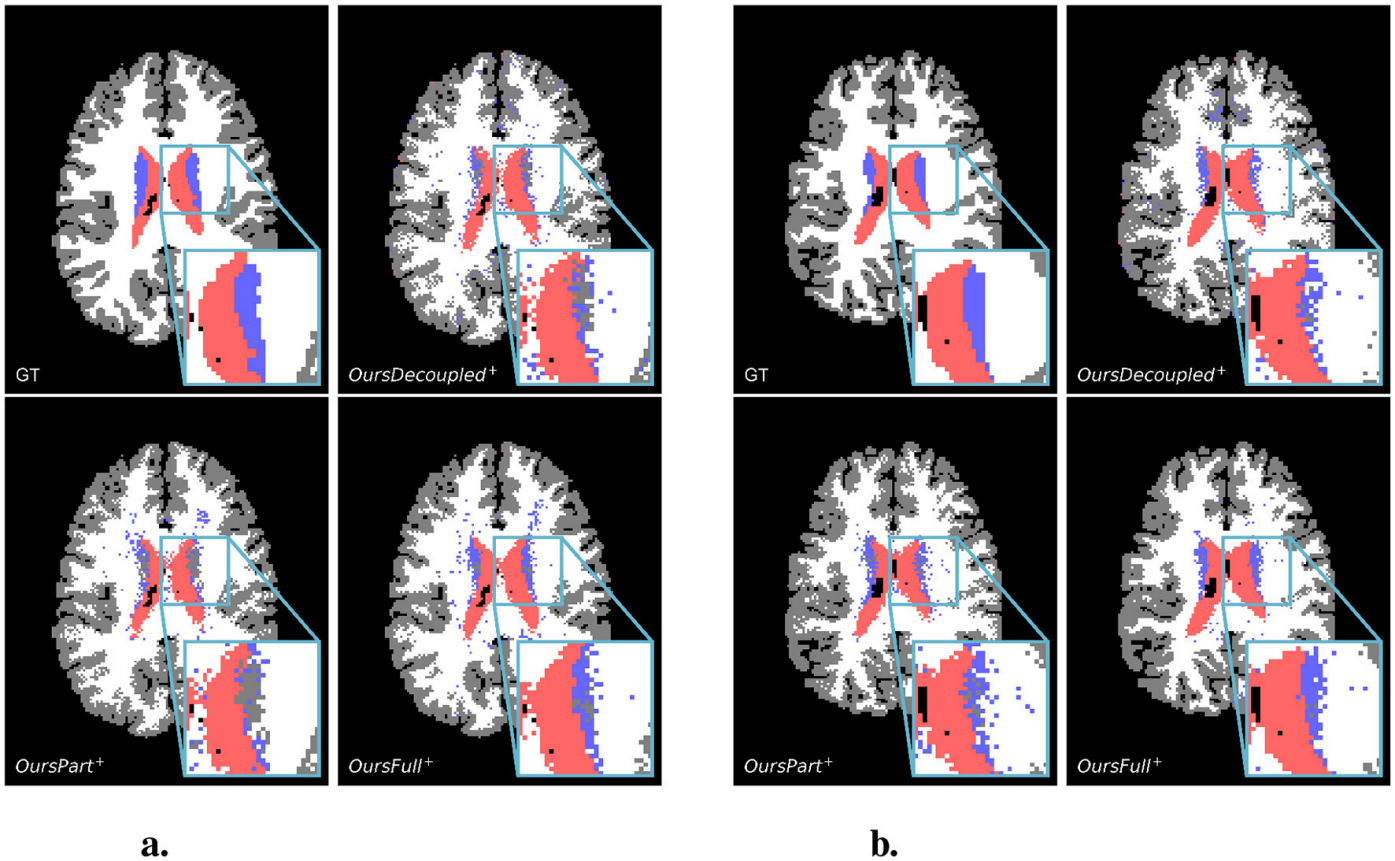
Showcases of zoom-in regions from predictions of the rotated test set. For both scan slices presented, from left to right, top to bottom, are the ground truth, prediction from OursDecoupled^+^, OursPart^+^, and OursFull^+^. The colors of different regions are the same as in Figure 6. **(A)** A test scan slice. **(B)** Another test scan slice.

Examples of predictions using the rotated test set can be found in Figure 6B. It is easily observed that the classical CNN does not generalize well to the data variation, while models with rotational symmetry (either *SO*(3), 𝕋^3^ × *SO*(3), or *SE*(3)) generate better results. However, it is also noticeable that for a challenging minority class, subcortical region, OursFull^+^ performs better than the others while other models with some rotational equivariance do not predict a concentrated subcortical region. Zoom-in examples can be found in Figure 7. Predictions from Baseline are omitted from Figure 7 since it does not have the same level of performance.

##### 4.3.7.1 Augmentation in training data vs. augmentation in testing data

**Figure 8:**
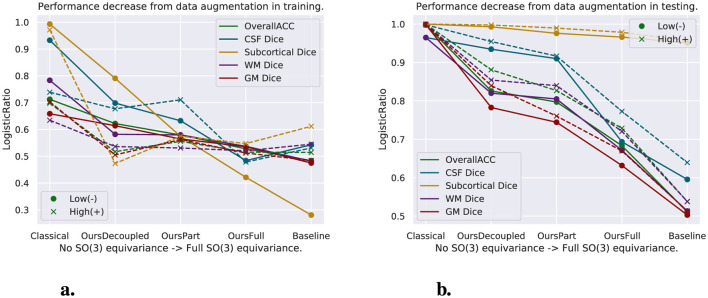
Logistic map of the ratio of two criteria to evaluate the proposed models. One criterion is for the models trained with augmented data compared to their counterparts trained with original data. For models trained both with original and augmented data, the left figure shows the decrease of test results while trained with data augmentation and tested with the original test set as shown in Tables 3–6. The second criterion is for the models trained with original data only. It is the decrease of performance while tested with rotated data, shown on the right figure. **(A)** Model performance decrease while trained with data augmentation. **(B)** Model performance decrease while applied with rotated test set.

We have experimented models trained with both the original training set and augmented training set, and models tested with both the original test set and randomly rotated test set. The random rotations applied to the test set can be seen as augmentation too. As was discussed above, data augmentation changes the distribution of the dataset, which creates inconsistency between the training and testing set. However, augmentation in the training set enables the models to see more data and thus even tested with the original test set, the performance of any model does not go far off, since the model has seen the type of data in the test set. The performance of models trained with data augmentation is worse than that of models trained with the original training set, though, due to the inconsistency of distributions between the training set and test set when only one of them is augmented. Figure 8A shows, for models tested with the original test set only, the decrease of model performance from models trained with the original training set to models trained with data augmentation. The *y*-axis shows the logistic map of the ratio of the performance decrease and is calculated by $L\left(x\right)=\frac{1}{e^{-\alpha x}}$ with α = 20, $x=\frac{C_{original}}{C_{augmented}}$, and *C*_*original*_ and *C*_*augmented*_ are the numbers indicating the performance (in this case, either dice score or accuracy as shown in the figure) of models tested with only the original test set but trained with the original (*C*_*original*_) or augmented (*C*_*augmented*_) training set. We can see from Figure 8A that the performance of the equivariant models we propose decrease less. This shows, from one perspective, the resistance of equivariant models to inconsistency of data distributions between training and testing data. On the other hand, having data augmentation only in the test set becomes a big problem for models without equivariance. Figure 8B shows, for models trained with the original training set only, the performance decrease from models tested with the original test set to those tested with rotated data. The *y*-axis values are calculated the same as the formula above, but the *C*_*original*_ and *C*_*augmented*_ become the numbers indicating the performance of models trained with the original training set only but tested with the original (*C*_*original*_) or rotated (*C*_*augmented*_) test set. We can see clearly from Figure 8B as well that the performance of classical CNN decreases the most using rotated data, and the decrease of performance goes down when we enforce more spatial equivariance in the model. Baseline models decrease the least, but again, the performance is limited due to the lack of information in ℝ^3^. Furthermore, the *SE*(3)-equivariance is implemented separately for the spatial and spherical parts and is with interpolation in the spatial part; thus, there are some errors introduced to it. Therefore, OursFull models always perform the best when there is variation in the test data.

##### 4.3.7.2 Rotational invariance for Type 1 discretization

Furthermore, we have also experimented with networks that have some rotational invariance but in the classical CNN setup - viewing the DWI images as *I*:ℝ^3^ → ℝ^*N*^. Taking the classical CNN setup we have in Section 4.2.1, we rotate the CNN kernels in each layer using the same rotations as in Section 4.2.4 to discretize *SO*(3). As was done above, we use the 60 rotations from the icosahedral symmetry group as well as only 12 of them (1 at each rotation axis) to act on the CNN kernels. In each layer, one rotation of the kernel is only convolved with the response of the corresponding rotation from the last layer; thus, this network is in fact 60 (or 12) independent networks, in which they share the same weights of different rotations. At the end, we take the average of the 60 (or 12) responses from all the rotations. With a small trial, we discovered that, as expected, even though this type of network does not perform as well as our spatial-directional GCNN as a whole, the performance decreases little in the full icosahedral group case with 60 rotations when tested with augmented data and decreases more when only a subset (12) of the group is used to rotate the kernels (see Table 11).

**Table 11:** Augmented CNN tested with original and rotated data.

**Rotations**	**Data type**	**CSF dice**	**Subcortical dice**	**WM dice**	**GM dice**	**Overall ACC**
90 − 5−5 − 5−*FC***, #Param 13539**						
Part(12)	Original	0.798 ± 0.058	0.425 ± 0.052	0.843 ± 0.01	0.875 ± 0.01	0.838 ± 0.011
	Rotated	0.71 ± 0.074	0.306 ± 0.042	0.755 ± 0.014	0.796 ± 0.014	0.75 ± 0.013
Full(60)	Original	0.754 ± 0.065	0.485 ± 0.059	0.823 ± 0.014	0.848 ± 0.02	0.818 ± 0.016
	Rotated	0.75 ± 0.063	0.479 ± 0.059	0.813 ± 0.013	0.838 ± 0.02	0.809 ± 0.016

This further demonstrates that having rotational equivariance in the model makes it much more robust to variance in the data - which, with no need of explanation, is inevitable when dealing with real-world raw data. Averaging rotational copies of a classical CNN achieves the goal of dealing with variance in data, but for non-linear data such as DWI, for which signals in voxels have some geometric structure, our full *SE*(3)-GCNN provides the best solution.

### 4.4 Comparison to state-of-the-art

We now compare our method to the approach of Müller et al. (2021). They used DWI data with q-space encoding in the diffusion part and the spatial part of the data is referred to as p-space, and these two parts of the data resemble the *S*^2^ and ℝ^3^ spaces in our formulation. We use the *b*-vectors from the HCP dataset as the input to the q-space. In their case, the input of the network is a whole DWI scan, not a series of extracted patches like we do, and we cannot fit an entire HCP scan into the model without exceeding the memory limit of a 24 GB GPU. After discussion and agreement with one of the authors (V. Golkov), we decided to use a modified architecture of their network to get an as fair as possible comparison: (1) we provide their network with patches of the same size as ours (7 × 7 × 7), but with DWI signals that are only normalized by *b*0 instead of interpolated spherical functions in each voxel like we did in our method. (2) The best performing model hyper-parameters they provided in the paper (with 4 and 5 layers in totals) are optimized for receptive fields that are much larger than ours, we use instead their 3-layer network, which has almost the same level of performance. (3) We have also disabled padding in their network to cancel biases introduced in the networks. After 3 *p*-spatial layers, the output of their network without padding has spatial dimensions 1 × 1 × 1. Their method and ours thus perform the same task: voxel-wise classification. We used the Focal Loss (Lin et al., 2018) using the same parameters as all the experiments above. We used the suggested structure of their network with fully connected layers in the radial basis, which reportedly has better performance than ones without them. To make the comparison fair, we use a network whose hyper-parameters are different from what was presented in Liu et al. (2022) such that the number of trainable parameters is similar to that of Müller et al. (2021).

#### 4.4.1 Network architectures

For Müller et al. (2021), we use the 1(*pq*)+1(*q*−*reduction*)+2(*p*) layer structure with the *TP*±1 basis presented in their paper and channels (5, 3, 0, 0), (5, 3, 0, 0), (10, 5, 0, 0), (4, 0, 0, 0) as presented in the Appendix section E.1 in their paper, except that we changed the output channel to 4 to fit our multiclass classification task and changed the *p*-space kernel sizes to 3 to ensure that the receptive field of the network is 7 × 7 × 7, as we discussed with the author. For our method, we use a *ReLU*(*lift*)−*ReLU*(*gconv*)−*ReLU*(*gconv*)−*project*−*FC* architecture such that there are three spatial layers as in Müller et al. (2021). With each layer split into 2, we use 10 − 10 − 20 − 40 − 20 − 10−*proj*.−4 as our layer structure such that we have similar numbers of parameters as Müller et al. (2021). Our method has 34964 parameters, while Müller et al. (2021) has 34,781 parameters.

#### 4.4.2 Results

The results are shown in Table 12. We can see that our method performs better than Müller et al. (2021). To test the equivariance of both methods, we again test both models with the randomly rotated test set as presented above, and the results can be found in Table 13.

**Table 12:** Statistics of results from both our method and Müller's method.

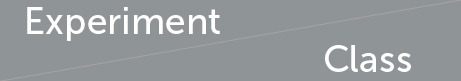	**CSF**	**Subcortical**	**WM**	**GM**	**Overall**
**Accuracy**					
Ours	**0.804**±0.073	**0.754**±0.033	**0.871**±0.018	**0.908**±0.011	**0.882**±0.008
Müller's	0.583 ± 0.123	0.442 ± 0.176	0.83 ± 0.036	0.834 ± 0.033	0.805 ± 0.015
**Dice score**					
Ours	**0.799**±0.053	**0.722**±0.034	**0.877**±0.008	**0.908**±0.006	
Müller's	0.655 ± 0.086	0.41 ± 0.105	0.813 ± 0.015	0.849 ± 0.016	

The bold values highlight the maximum value in the column.

**Table 13:** Statistics of results from both our method and Müller's method tested with rotated test set.

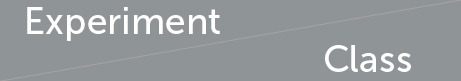	**CSF**	**Subcortical**	**WM**	**GM**	**Overall**
**Accuracy**					
Ours	**0.725**±0.083	**0.596**±0.036	**0.834**±0.02	**0.874**±0.013	**0.838**±0.008
Müller's	0.445 ± 0.1	0.337 ± 0.146	0.823 ± 0.036	0.789 ± 0.031	0.771 ± 0.014
**Dice score**					
Ours	**0.742**±0.067	**0.593**±0.032	**0.832**±0.009	**0.875**±0.006	
Müller's	0.426 ± 0.055	0.343 ± 0.104	0.787 ± 0.015	0.813 ± 0.015	

The bold values highlight the maximum value in the column.

**Figure 9:**
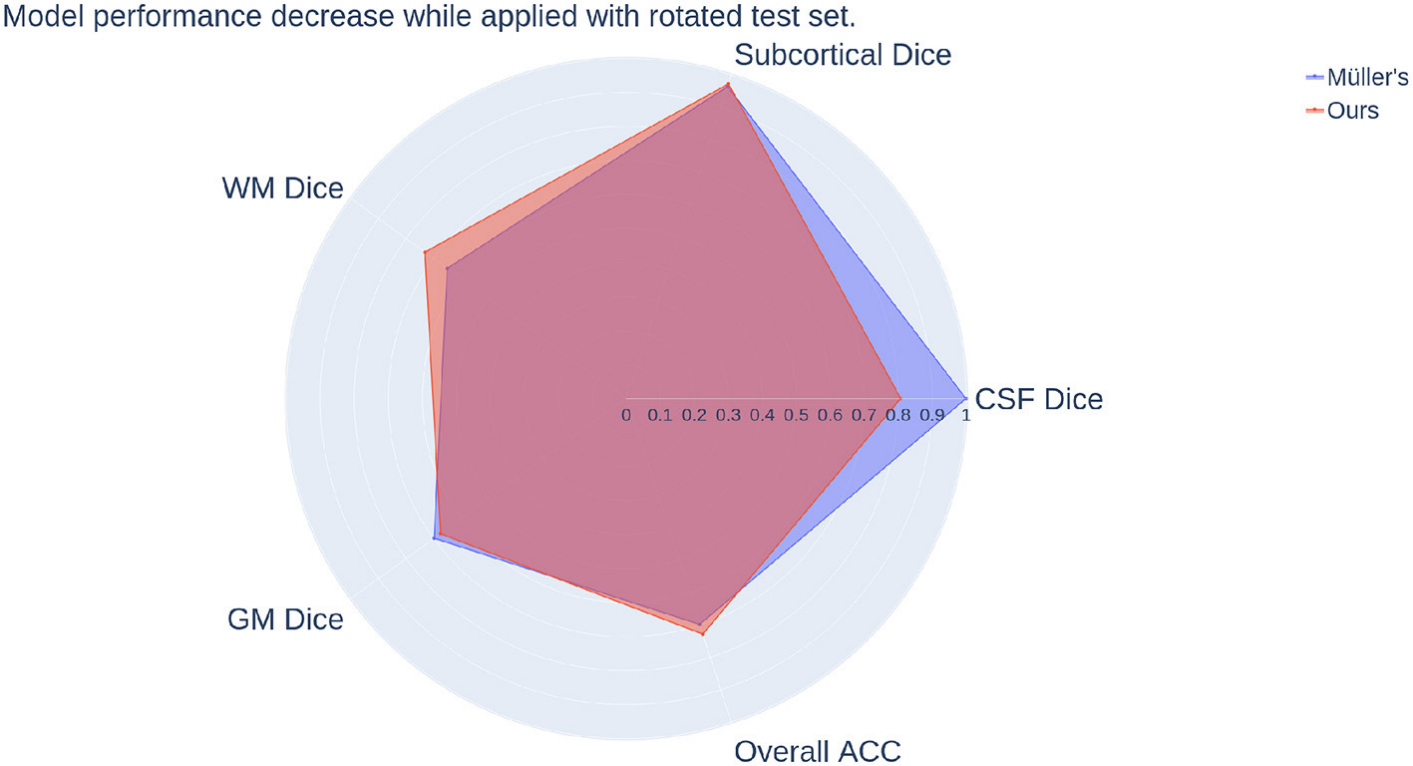
Comparison of model performance decrease while applied with rotated test set between our method and Müller's. The radial axis indicates the decrease, and it is the logistic map of the ratio calculated by the same scheme used in Figure 8.

We can see from the numbers that the performance of Müller et al. (2021) does not drop much either while tested with unseen rotated test set, similar to our method. As we can see from Figure 9, overall, Müller et al. (2021) lost less in percentage of the Dice scores of Subcortical, White matter, and overall accuracy but more in CSF Dice score. Both equivariant methods are more resistant to variations in the distributions of the training and test set than the non-equivariant models presented above. Moreover, since the overall performance decrease of Müller et al. (2021) while tested with rotated data is lower than our fully equivariant model, Müller et al. (2021) actually has better equivariance than all models we presented even though their prediction accuracies and dice scores are lower.

### 4.5 Comparison to non-NN spherical harmonics feature classification

Following the method described in their paper, we extracted spherical harmonic features from each voxel of *b*−1000 DWIs and used SVMs for classification. Both one-vs-one and one-vs-all SVM configurations were applied to evaluate their comparative effectiveness in handling multiclass data. To normalize features, we experimented with both standard and min-max normalization methods. The performance of each setup was assessed using accuracy and Dice score metrics, consistent with the evaluation metrics for our proposed method. The results are shown in Table 14.

**Table 14:** Results for all models from Schnell et al. (2009) for *b* = 1000.

**Norm, metric**	**CSF**	**Subcortical**	**WM**	**GM**	**Overall**
**OVO**					
Standard, ACC	0.772 ± 0.007	0.007 ± 0.000	0.918 ± 0.005	0.538 ± 0.057	0.678 ± 0.000
Standard, Dice	0.732 ± 0.006	0.014 ± 0.000	0.728 ± 0.003	0.627 ± 0.025	
Minmax, ACC	0.785 ± 0.006	0.003 ± 0.000	0.906 ± 0.007	0.576 ± 0.075	0.692 ± 0.000
Minmax, Dice	0.729 ± 0.007	0.005 ± 0.000	0.739 ± 0.004	0.642 ± 0.034	
**OVR**					
Standard, ACC	0.695 ± 0.011	0.000 ± 0.000	0.920 ± 0.004	0.570 ± 0.044	0.693 ± 0.000
Standard, Dice	0.737 ± 0.006	0.000 ± 0.000	0.738 ± 0.003	0.659 ± 0.018	
Minmax, ACC	0.724 ± 0.010	0.000 ± 0.000	0.920 ± 0.006	0.554 ± 0.069	0.686 ± 0.000
Minmax, Dice	0.740 ± 0.006	0.000 ± 0.000	0.736 ± 0.004	0.633 ± 0.031	

Both One-vs.-One (OVO) and One-vs.-Rest (OVR) models using Standard and Minmax normalizations are presented. The values shown in the table are the mean and standard deviation of the chosen metrics, and three decimals are used; therefore, some very small values are shown as 0.

As shown in Table 14, the performance of the method from Schnell et al. (2009) is significantly lower than that of our proposed approach. In particular, for the challenging class–the subcortical region–the model showed minimal recognition capability. This result is expected as the rotation-invariant features derived independently from individual voxels inherently disregard the spatial relationships among voxels, which undermines model robustness. Furthermore, our *SO*(3) models, which similarly do not incorporate spatial voxel connectivity, nonetheless outperform the method in Schnell et al. (2009), underscoring the robustness and stability introduced by the equivariant convolutions within our model.

## 5 Discussion

The resistance to data variation that has been shown by our fully equivariant network was demonstrated on synthetically augmented data - with 90-degree rotations. Even though this synthetic augmentation did not cost any loss of signals or any interpolation-caused inaccuracy, it is desirable to verify the robustness of more complex group actions in CNNs using data with real-world variations (e.g., subjects scanned in different positions, affine variations in shapes). Acquiring this type of data is another challenge. On the other hand, data augmentation seems to be very robust against the variations in the rotated test set. However, this is because the augmentations applied in the training set and the test set are identical, and they modeled exactly the same distribution in the data. Our proposed equivariant methods deal with inconsistent distributions between the training set and the test set much better, which is usually the case in real world. In addition, our method outperforms (Müller et al., 2021) with the same amount of information given to the models. Even though both methods show similar resistance to variations in the distributions of the training and test set, our model has a more light-weight implementation using regular group representation with separable kernels. Furthermore, the experiments we conducted using Schnell et al. (2009) have shown the power of equivariant learning in non-Euclidean spaces. Using rotation-invariant features as in Schnell et al. (2009) is beneficial in terms of getting consistent features from spherical functions, regardless of the orientation. However, extracting invariant features from the very beginning also discards potentially valuable orientational information that is implicitly embedded in the data, and discarding spatial information completely severely weakens the capability of the model. This is easily shown by the fact that our *SO*(3) models that also discard spatial relationships outperform (Schnell et al., 2009).

In conclusion, we presented a systematic study of GCNNs of various group actions with the application to DWI segmentation. We interpreted images of DWI scans (*I*:ℝ^3^×*S*^2^ → ℝ) as functions in the homogeneous spaces of groups with different complexities of symmetries and provided a detailed analysis of how different levels of complexities of these symmetries impact the performance of the network. It is shown from the models OursDecoupled and OursFull that whether or not more complex transformations should be imposed in the model is not always a clear-cut, since while tested on the original test set, OursDecoupled has a slightly better performance. OursDecoupled incorporates a mathematically well-defined, but physically impossible group action, yet it is computed more cheaply, while OursFull incorporates the SE(3) action, which corresponds to the expected physical transformations of the data. And, under any physically realistic turbulence in the test data resulting in unseen distributions, adding to the model possible transformations of the data (to the limit of their discretizations) provides a more stable performance. Therefore, we emphasize the importance of imposing the full roto-translation transformations in models as it is the kind that appears in the data. From the experiments, we conclude that (1) exploiting the spatial-directional interactions in the data is crucial for efficient learning of the features; (2) incorporating complex group actions of 3D rigid motions—SE(3)—might not be essential for highly aligned and preprocessed data such as the human connectome project (HCP) (Van Essen et al., 2013), but it shows significantly higher resistance to variations in data. For real-world raw data in which the positions of subjects are not perfectly aligned as in Van Essen et al. (2013), our proposal shows significant potential.

## Data Availability

The original contributions presented in the study are included in the article/Supplementary material, further inquiries can be directed to the corresponding author.
