# Supplementary material for: SE(3) group convolutional neural networks and a study on group convolutions and equivariance for DWI segmentation
Source: Front Artif Intell. 2025 Feb 28;8:1369717. doi: 10.3389/frai.2025.1369717 (PMC11906406; doi:10.3389/frai.2025.1369717)
Supplement: Supplementary file 1 [file Data_Sheet_1.pdf]

Appendix to: $SE(3)$ Group Convolutional Neural Networks and a Study on Group Convolutions and Equivariance for DWI Segmentation: Recall on Transformation Groups

1 Transformation Groups

In this section we recall elements of group theory, transformation groups and group actions.

1.1 Groups and Lie Groups

1.1.1 Groups.

A group G is a set with a distinguished element e, called the neutral, or identity element of the group, and endowed with two operations: the product $G \times G \to G$, $(g_1, g_2) \mapsto g_1 \cdot g_2 \in G$, and an inversion, $g \mapsto g^{-1} \in G$, that satisfy the following axioms.

1. Associativity. For $g_1, g_2, g_3 \in G$, $(g_1 \cdot g_2) \cdot g_3 = g_1 \cdot (g_2 \cdot g_3)$.
2. Inversion: $g \cdot g^{-1} = g^{-1} \cdot g = e$.
3. Neutral and product: $g \cdot e = e \cdot g = g$.

A subset $H \subset G$ is a subgroup if H is closed for the group operations of G: if $h_1, h_2 \in H$, $h_1 \cdot h_2 \in H$ and $h_1^{-1} \in H$, so that H is a group with these operations. In particular, if $h \in H$, then $e = h \cdot h^{-1} \in H$ is the neutral element of H.

$\mathbb{R}^n$ is a group for addition, $GL(n)$ is the group of invertible transformations of $\mathbb{R}^n$ (or the group of invertible matrices in $\mathbb{R}^{n \times n}$). If G and H are groups, we can for the product group $G \times H$ whose underlying set is the product set, and for which operations are defined element-wise: if $(g_1, h_1), (g_2, h_2) \in G \times H$, $(g_1, h_1) \cdot (g_2, h_2) = (g1_1 \cdot g_2, h_1 \cdot h_2)$ and $(g, h)^{-1} = (g^{-1}, h^{-1})$.

There are more complicated products, called *semi-direct* products, a symbol $\ltimes$ is used instead of $\times$. We are mainly interested in the case where H is a subgroup of $GL(n)$ and $\mathbb{T}^n$ is the group of translations of $\mathbb{R}^n$ (basically $\mathbb{R}^n$ under

another name). The semi-direct product $H \ltimes \mathbb{T}_n$ is, as a set, the product set, but the group product is more complicated. for $M_1, M_2 \in GL(n)$, $\vec{t_1}, \vec{t_2} \in \mathbb{T}^n$,

$$(M_1, \vec{t_1}) \cdot (M_2, \vec{t_2}) = (M_1 M_2, \vec{t_1} + M_1 \vec{t_2}).$$

When $H = SO(n)$, the group of rotations of $\mathbb{R}^n$, $H \ltimes \mathbb{T}^n = SE(n)$, the special Euclidean group.

1.1.2 Quotient group.

Let $H \subset G$ be a subgroup. The left cosets of H are the subsets $gH = \{gh, h \in H\}$. Two left cosets $g_1 H$ and $g_2 H$ are equal if and only if $g_2^{-1} g_1 \in H$. Two distinct cosets are *disjoint*. The coset set $\{gH, g \in G\}$ is the (left) quotient space of G by H, denoted by G/H.

1.1.3 Lie Groups.

A Lie group G is a group, whose underlying set is a manifold, such that the product and inversion mapping are smooth for this structure. A vector space is a Lie group, the group of orthogonal transforms $O(n)$ and its subgroup of rotations $SO(n)$ is. $\mathbb{T}^n$ and $SE(n)$ are too.

1.2 Actions and Representations

1.2.1 Group Action.

Given a set $\mathcal{M}$, a left G-*action* on M is a mapping $\alpha : G \times \mathcal{M} \to \mathcal{M}$, $(g, m) \mapsto g \cdot m$, which satisfy the following axioms

1. $e \cdot m = m$ for all m in m, i.e., the mapping $m \mapsto e \cdot m$ is the identity,

2. $g_1 \cdot (g_2 \cdot m) = (g_1 g_2) \cdot m$.

Fixing $g \in G$, the mapping

$$g \cdot : \mathcal{M} \to \mathcal{M}, \quad , m \to g \cdot m \tag{1.1}$$

is a bijection, whose inverse is $g^{-1} \cdot$. With this action, $\mathcal{M}$ is called a G-space. G is itself a G space in several ways: the left multiplication $h \cdot : G \to G$, $g \mapsto hg$ is left action. Conjugation is another one: $c_h : g \mapsto hgh^{-1}$ is also a left action. One talks of Lie-group action when $\mathcal{M}$ is a manifold and the action operations above are smooth.

The *trivial* action of a group G on a set $\mathcal{M}$ is the action $g \cdot m = m$ for any $g \in G$, $m \in \mathcal{M}$. This action does not change anything but allows to define invariance as a special case of equivariance.

1.2.2 Orbits and Stabilizers.

With $\mathcal{M}$ a G-space, and $m \in \mathcal{M}$, the orbit $G.m$ of m is the set $\{g \cdot m, g \in G\} \subset \mathcal{M}$, this is the set made of all the possible transformations of m by elements of G. The stabilizer G_m of m, also called *isotropy subgroup, or little group* of m, is the set of G-*symmetries* of m, i.e., $G_m = \{g \in G, g \cdot m = m\}$.

Two distinct orbits are disjoint and $\mathcal{M}$ is a disjoint union of its orbits.

1.2.3 Homogeneous spaces

When there is only one orbit, i.e., if given m_1 and m_2 in $\mathcal{M}$, there exists $g \in G$ with $m_1 = g \cdot m_1$, $\mathcal{M}$ is said to be a homogeneous space of G. If the transformation g which sends m_1 to m_2 is always unique, $\mathcal{M}$ is a *principal homogeneous space* of G. Choosing a *base point* $m_0 \in \mathcal{M}$, one obtains an identification $G \simeq (\mathcal{M}, m_0), g \mapsto gm_0$ In general, if $\mathcal{N}$ is a G- homogeneous space, $n_0 \in \mathcal{N}$ a *base point*, and G_{n_0} is its stabilizer, there is an identification $G/G_{n_0} \simeq \mathcal{N}$ given by

$$gG_{n_0} \in G/G_{n_0} \mapsto g \cdot n_0. \tag{1.2}$$

This is well-defined as, if $g' \in gG_{n_0}$, then $g' = gh$, for a $h \in G_{n_0}$ and $g' \cdot n_0 = gh \cdot n_0 = g \cdot (h \cdot n_0) = gn_0$ since $h \in G_{n_0}$. The coset gG_{n_0} is called the *fiber above* n. This depends of course on the choice of the base point n_0. This identification of the homogeneous space with a quotient of G is at the core of the definition of our projection layers.

1.2.4 Equivariant and invariant mappings.

A mapping $f : \mathcal{M} \to \mathcal{N}$ between G-spaces $\mathcal{M}$ and $\mathcal{N}$ is called *equivariant* if $f(g \cdot m) = g \cdot f(m)$. A mapping $f : \mathcal{M} \to \mathcal{N}$, where $\mathcal{M}$ is a G-space, is*invariant* if $f(g \cdot m) = f(m)$, for $\forall g \in G$. This is the same as saying that $f : \mathcal{M} \to \mathcal{N}$ is equivariant when $\mathcal{N}$ is endowed with the trivial G-action, as in that case $g.f(m) = f(m)$.

1.2.5 Representations and left Regular Representations

Assume V is a vector space with a G-action. If, for each $g \in G$, the transformation (1.1) is *linear*, the action is called a *linear representation* of G. The main example in this work is the *left regular representation.* If G acts on a space $\mathcal{M}$, it will act on functions on $\mathcal{M}$, by transforming - "translating" their argument. If $f : \mathcal{M} \to \mathbb{R}$, one defines $L_g f$ as the function $m \mapsto f(g^{-1}m)$. When $V = L^2(\mathcal{M})$, and $f \in V$, the transformation $f \mapsto L_g f$ is linear, L is the *left regular representation* on V.
